# Midterm Outcomes of Transcatheter Edge-to-Edge Repair for Primary Mitral Regurgitation According to Anatomical Characteristics

**DOI:** 10.1016/j.shj.2025.100763

**Published:** 2025-11-17

**Authors:** Daryoush Samim, Caroline Chong-Nguyen, Yannick Hausammann, Mischa Külling, Oliver Gaemperli, Roberto Corti, Joanna Bartkowiak, Daijiro Tomii, Domenico Angellotti, Nicolas Brugger, Thomas Pilgrim, Patric Biaggi, Fabien Praz, Peter Martin Wenaweser

**Affiliations:** a Department of Cardiology, Inselspital, Bern University Hospital, Bern, Switzerland; b Clinical Trial Service Unit and Epidemiological Studies Unit, Nuffield Department of Population Health, University of Oxford, Oxford, UK; c HerzKlinik Hirslanden Zürich, Zürich, Switzerland; d Faculty of Medicine, University of Zürich, Zürich, Switzerland; e Department of Advanced Biomedical Sciences, University of Naples Federico II, Naples, Italy

**Keywords:** Anatomy, Echocardiography, Mitral regurgitation, Mitral valve, Prognosis, Transcatheter edge-to-edge repair

## Abstract

**Background:**

Mitral transcatheter edge-to-edge repair (M-TEER) is an established option for high-risk primary mitral regurgitation (PMR) patients, but data on the impact of anatomical complexity on prognosis are scarce and conflicting.

**Objectives:**

The aims of this study were to characterize patients with severe PMR undergoing M-TEER, assess mid-term prognosis after M-TEER, and identify prognostic factors based on PMR mechanism.

**Methods:**

Data from symptomatic PMR patients with severe PMR treated with M-TEER between July 2013 and October 2023 at two Swiss centers were collected retrospectively until 2017 and prospectively thereafter. Patients were categorized by lesion type: A2-P2 prolapse/flail vs. non-A2-P2 prolapse/flail. A subset was classified by mitral valve (MV) anatomical complexity (defined by the presence of ≥1 of the following: ≥moderate calcifications, Barlow’s disease, multiple prolapses, or commissural prolapses). Cox regression identified predictors of 1-year all-cause mortality.

**Results:**

Among 315 patients (mean age 82.2 ± 6.3 years, 46.3% female, European System for Cardiac Operative Risk Evaluation II 5.1% ± 4.1%) followed for a median (interquartile range [IQR]) of 13 months (5-33), technical success was 93.0%. Compared with the non-A2-P2 prolapse/flail group (n = 186), the A2-P2 prolapse/flail group (n = 129) had better echocardiographic outcomes at discharge (residual mitral regurgitation [MR] ≤ 1+: 70.5 vs. 60.4%; *p* = 0.031) and superior symptomatic improvement at 1 year (New York Heart Association class ≤ II: 91.4 vs. 74.5%; *p* = 0.017) but similar 1-year all-cause mortality (15.1 vs. 18.8%; *p* = 0.492). Among patients classified by MV anatomical complexity (n = 143), patients with complex MV anatomy (n = 68) had a higher mortality at a median (IQR) follow-up of 22 months (9-36) compared to those with noncomplex MV anatomy (n = 75) (51.5 vs. 34.7%; *p* = 0.042). Multivariate analysis identified complex MV anatomy and severe renal failure as predictors of 1-year all-cause mortality.

**Conclusions:**

MV anatomical characteristics have a significant influence on symptomatic improvement and all-cause mortality at 1 year and should be carefully considered during the selection of PMR patients for M-TEER.

## Highlights

• Mitral transcatheter edge-to-edge repair (M-TEER) is an established treatment option in patients with primary mitral regurgitation (PMR) at high or prohibitive surgical risk. Data regarding the impact of anatomical complexity on prognosis are scarce and conflicting.
• Our study on M-TEER for PMR according to anatomy is the first to include patients treated over a period of more than 10 years. We demonstrate that, regardless of the underlying mitral regurgitation (MR) lesion, M-TEER is a safe procedure that effectively reduces PMR and alleviates symptoms with a high technical success rate (>90%).
• In addition to clinical comorbidities, our data show that mitral valve (MV) anatomical characteristics are associated with outcomes and should thus be considered in the selection of PMR patients undergoing M-TEER.
• Future larger-scale and mechanistic studies are needed to address long-term outcomes of M-TEER according to anatomy, focus on PMR patients with complex MV anatomy, and confirm the durability and safety of this treatment approach.

## Graphical abstract

Graphical Abstract: Midterm Outcomes of Trantscatheter Edge-to-Edge Repair for Primary Mitral Regurgitation According to Anatomical Characteristics. MV, mitral valve; MR, mitral regurgitation; M-TEER, mitral transcatheter edge-to-edge repair; PMR, primary mitral regurgitation.

**Figure:**
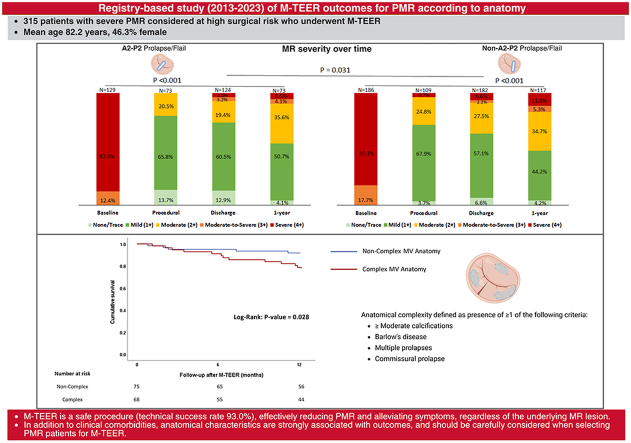

## Introduction

Primary mitral regurgitation (PMR) affects more than 24 million people worldwide.^1^ Population-based studies in high-income countries have suggested that clinically significant PMR (moderate or higher grade) is found in 2% of patients and is more common in females.^2^^,^^3^ The prevalence increases with age in both sexes and reaches 4.8% in patients >75 years old, a prevalence similar to that of aortic stenosis in this age category.^2^^,^^3^ Left untreated, severe PMR may lead to heart failure (HF) with left ventricular dysfunction, reduced cardiac output, and pulmonary congestion, affecting quality of life and survival.^4^

Surgery represents the standard of care owing to favorable effectiveness and long-term results of mitral valve (MV) repair in observational studies and should be preferred over replacement when valve anatomy is suitable and perioperative risk is acceptable.^5^^,^^6^ In PMR patients at high or prohibitive surgical risk according to Heart Team assessment and for whom the procedure is not considered futile, current consensus guidelines recommend mitral transcatheter edge-to-edge repair (M-TEER).^7^^,^^8^ M-TEER has been used for more than 20 years in patients suffering from PMR, and the safety and effectiveness of the technique have been demonstrated in randomized trials and observational studies.^9^, ^10^, ^11^ Nowadays, patients with growing anatomical complexity going far beyond the initially defined EVEREST II (Endovascular Valve Edge-to-Edge Repair Study) criteria^12^ are selected by increasingly experienced operators and imaging teams. However, clinical outcomes according to the anatomy and etiology of PMR have rarely been reported^13^^,^^14^ and are yet to be clearly defined.

This study aimed to characterize patients with severe PMR who underwent M-TEER, assess mid-term prognosis after M-TEER, and identify prognostic factors according to PMR etiology and anatomy.

## Methods

### Study Design and Patients’ Selection

Clinical, echocardiographic, and biological data from symptomatic patients with severe PMR who underwent M-TEER using the MitraClip system (Abbott Vascular, Abbott Park, Illinois, USA) or PASCAL system (PASCAL, Edwards Lifesciences, Irvine, USA) between July 2013 and October 2023 at two high-volume centers in Switzerland (University Hospital of Bern & HerzKlinik Hirslanden Zürich) were collected retrospectively until 2017 and prospectively afterward into a dedicated registry. The selection criterion for this study was as follows: severe PMR with indication for M-TEER according to the Heart Team.

Each patient was evaluated by a multidisciplinary Heart Team composed of HF specialists, interventional cardiologists, cardiac surgeons with expertise in MV replacement, multimodality imaging specialists, and cardiac anesthesiologists. The interdisciplinary Heart Team discussed each patient and opted for M-TEER as the therapy of choice. The choice of device was at the operator's discretion. Patients underwent M-TEER and were treated according to recommendations^7^^,^^8^^,^^15^, ^16^, ^17^ at the corresponding time of intervention and according to each center’s standard of care. Echocardiographic evaluation prior to and after M-TEER was performed by experienced physicians at each center according to recent recommendations.^18^

### Study Variables

Clinical baseline characteristics included demographic, laboratory (creatinine, n-terminal pro-B type natriuretic peptide, and hemoglobin), and medical data as well as relevant comorbidities. HF symptoms were assessed according to the New York Heart Association (NYHA) functional class. Severity and etiology of MR were assessed by each site using an integrative approach to grade MR severity based on an established five-grade system (none/trace, 1+, 2+, 3+, 4+).^19^, ^20^, ^21^ Primary MR was defined as MR due to abnormal MV leaflets, leaflet calcification, and/or chordae that may be associated with leaflet prolapse or flail. As a first step, patients were classified according to the characteristics of the lesion causing PMR: A2-P2 prolapse/flail vs. non-A2-P2 prolapse/flail. As a second step, a subgroup analysis was performed according to MV anatomical complexity based on preinterventional transesophageal echocardiography (TEE) three-dimensional (3D) images by a dedicated core laboratory at the University Hospital of Bern while maintaining blinding on patient information and outcomes. Complex MV anatomy was defined as the presence of ≥1 of the following criteria: moderate or severe calcifications, Barlow’s disease, multiple prolapses, or commissural prolapse.^10^^,^^12^^,^^22^, ^23^, ^24^ Echocardiographic assessment of the whole cohort also included left and right ventricular (RV) function and dimensions, left atrial volume index biplane, and mean transvalvular MV pressure gradient (mean MV inflow gradient). Baseline hemodynamic measurements obtained by left and right cardiac catheterization were collected.

### Outcomes

Study endpoints comprise technical success and key clinical endpoints according to the Mitral Valve Academic Research Consortium^25^^,^^26^ at discharge, 1 year, and last follow-up after M-TEER. Starting in 2017, a standardized 1-year clinical follow-up visit was planned. One-year mortality was assessed using the patient’s medical records as well as the national registry of deaths. For all patients alive, the date of last contact was considered. Echocardiographic outcomes (residual MR severity, single leaflet device attachment [SLDA]) were assessed by transthoracic echocardiography or TEE.

### Statistical Analysis

Statistical analyses were performed using RStudio version 2025.05.0 and SPSS version 25.0 for Windows. Results are expressed as absolute numbers and/or percentages for categorical variables and mean (± standard deviation [SD]) or median (interquartile range [IQR]) for continuous variables. Comparisons were performed using the χ^2^ test or Fisher exact test for categorical variables and Student’s *t*-test or Kruskal-Wallis test for continuous variables. Independent groups were compared using the Mann-Whitney *U* test, and changes in MR severity over time within the same patients were analyzed using Friedman’s test or Wilcoxon signed-rank test as appropriate. A multivariable Cox regression analysis was performed to identify predictors of 1-year mortality after M-TEER with results expressed as hazard ratios (HRs) and 95% CIs. Variables with *p* < 0.05 in univariate analyses at 1 year, as well as those previously associated with mortality in the literature, were entered into the multivariable model after assessing and excluding collinearity. We used the Kaplan-Meier method to estimate survival up to 1 year after M-TEER according to MV anatomical complexity.

### Ethical Statement

The study was approved by each center's local ethics committee and is in line with the principles outlined in the Declaration of Helsinki. All data are site-reported, and patients gave their consent.

## Results

The study included 315 consecutive patients with symptomatic PMR who underwent M-TEER at 2 Swiss centers.

### Baseline Clinical Characteristics

The baseline characteristics are summarized in Table 1. The mean age was 82.2 ± 6.3 years at the time of M-TEER, and A2-P2 prolapse/flail patients (n = 129) were slightly older than non-A2-P2 prolapse/flail patients (n = 186) (mean age 83.5 years ±5.5 vs. 81.3 years ±6.8; *p* = 0.003). Other baseline clinical characteristics were similar in both groups. About half the patients (146/315, 46.3%) were female. Patients were considered at intermediate and high surgical risk for MV replacement (European System for Cardiac Operative Risk Evaluation II 5.1% ±4.1 and Society of Thoracic Surgeons score 4.3% ±3.9). The prevalence of comorbidities was relevant: arterial hypertension 71.7% (226/315), coronary artery disease 36.8% (116/315), atrial fibrillation 51.7% (163/315), and anemia 46.3% (146/315). Two-thirds (214/315, 67.9%) of the patients were severely symptomatic (NYHA functional class ≥ III) despite a median furosemide equivalence dose of 40 mg (20-80). Pressures measured by left and right heart catheterization are summarized in Supplementary Table 1. Four out of five patients (106/133, 79.7%) had pulmonary hypertension (mean pulmonary arterial pressure ≥ 20 mmHg confirmed by invasive hemodynamic assessment) prior to M-TEER.

**Table 1:** Baseline characteristics

	All patients N = 315	A2-P2 prolapse/flail N = 129	Non-A2-P2 prolapse/flail N = 186	*p* value
Age at M-TEER (y)	82.2 ± 6.3	83.5 ± 5.5	81.3 ± 6.8	**0.003**
Female (%)	146 (46.3)	58 (45.0)	88 (47.3)	0.681
EuroSCORE II, %	5.1 ± 4.1	5.0 ± 4.1	5.1 ± 4.2	0.941
STS score for MV replacement, %	4.3 ± 3.9	4.2 ± 3.0	4.5 ± 4.4	0.656
BMI (kg/m2)	24.4 ± 4.7	24.1 ± 4.3	24.5 ± 5.0	0.469
Obesity (BMI ≥30 kg/m2)	35 (11.1)	14 (10.9)	21 (11.3)	0.903
Arterial hypertension	226 (71.7)	94 (72.9)	132 (71.4)	0.768
Severe renal failure (eGFR <30 ml/min/1.73 m2)	52 (16.5)	23 (17.8)	29 (15.6)	0.599
Diabetes	36 (11.4)	17 (13.2)	19 (10.2)	0.416
Dyslipidemia	117 (37.4)	47 (36.4)	70 (37.6)	0.837
History of malignancy	56 (17.8)	24 (18.6)	32 (17.2)	0.935
Atrial fibrillation	163 (51.7)	64 (49.6)	99 (53.2)	0.528
Chronic obstructive pulmonary disease	31 (9.8)	9 (7.0)	22 (11.8)	0.155
Coronary artery disease	116 (36.8)	47 (36.4)	69 (37.1)	0.905
Prior myocardial infarction	29 (9.2)	11 (8.5)	18 (9.7)	0.728
Prior percutaneous coronary intervention	83 (26.3)	35 (27.1)	48 (25.8)	0.793
History of stroke	31 (9.8)	14 (10.9)	17 (9.1)	0.616
Pulmonary∗ hypertension (mPAP ≥20 mmHg) (N = 133, 51 in A2-P2 and 82 in non-A2-P2)	106 (79.7)	44 (86.3)	62 (75.6)	0.137
Anemia (female: Hb < 120 g/L; male: Hb < 130 g/L)	146 (46.3)	62 (48.1)	84 (45.2)	0.612
Previous TAVI	8 (2.5)	3 (3.8)	5 (3.7)	0.964
Previous SAVR	11 (3.5)	4 (5.1)	7 (5.1)	0.979
Previous surgical MV repair	7 (2.2)	3 (2.3)	4 (2.2)	0.917
Previous CABG	25 (7.9)	10 (7.8)	15 (8.1)	0.920
Heart failure hospitalization in the last 12 mo prior to M-TEER (N = 291, 53 in A2-P2 and 90 in non-A2-P2)	41 (14.1)	16 (30.2)	25 (27.8)	0.758
NYHA functional class				0.467
I	30 (9.5)	16 (12.4)	14 (7.5)	
II	71 (22.5)	26 (20.2)	45 (24.2)	
III	179 (56.8)	72 (55.8)	107 (57.5)	
IV	35 (11.1)	15 (11.6)	20 (10.8)	
eGFR (ml/min)	49 [37-66]	48 [36-64]	48 [37-68]	0.327
Creatinine (μmol/L)	96 [80-127]	97 [79-134]	95 [80-125]	0.504
NT-proBNP (pg/ml)	2460 [931-4960]	3091 [1267-6556]	1961 [827-4143]	**0.022**
Renin-angiotensin system inhibitor				0.563
ACE	109 (34.6)	46 (35.7)	63 (33.9)	
ARB	98 (31.1)	37 (28.7)	61 (32.8)	
Sacubitril-valsartan	5 (1.6)	1 (0.8)	4 (2.2)	
MRA	32 (10.1)	15 (11.6)	17 (9.1)	0.468
B-blocker	185 (58.7)	74 (57.4)	111 (59.7)	0.691
Diuretics (excluding MRA)				0.569
One agent	240 (76.2)	98 (76.0)	142 (76.3)	
Two agents	7 (2.2)	1 (0.8)	6 (3.2)	
Loop diuretics dose Furosemide equivalence dose (mg)	40 [20-80]	40 [20-80]	40 [20-80]	0.918

Results are expressed as absolute number (percentage) for categorical variables and mean ±SD or median [interquartile range] for continuous variables.

Abbreviations: ACE, angiotensin-converting enzyme; ARB, angiotensin receptor blocker; BMI, body mass index; CABG, coronary artery bypass graft; eGFR, estimated glomerular filtration rate; EuroSCORE II, European System for Cardiac Operative Risk Evaluation II; Hb, hemoglobin; mPAP, mean pulmonary artery pressure; MRA, mineralocorticoid receptor antagonist; M-TEER, mitral transcatheter edge-to-edge repair; MV, mitral valve; NT-proBNP, N-terminal pro–B-type natriuretic peptide; NYHA, New York Heart Association; SAVR, surgical aortic valve replacement; STS, Society of Thoracic Surgeons; TAVI, transcatheter aortic valve implantation.

∗ mPAP measured invasively.

### Echocardiographic Characteristics Prior to M-TEER

Echocardiographic data prior to M-TEER are summarized in Table 2. The vast majority (266/315, 84.4%) of patients had severe (4+) PMR, and a minority had moderate-to-severe (3+) PMR (49/315, 15.6%). The most common pathology causing PMR was flail (146/291, 50.2%), followed by prolapse (102/291, 35.1%) and leaflet restriction (33/291, 11.3%). There were no differences between groups regarding baseline MV area (MVA) and mean MV pressure gradient. Most patients had preserved left ventricular systolic ejection fraction (246/315, 81.5%) with dilated left ventricle (mean indexed left ventricular end-diastolic volume 71 ± 27 ml/m2) and left atrium (mean biplane left atrial volume index 74 ± 30 ml/m2). Overall, RV longitudinal function was preserved (mean tricuspid annular plane systolic excursion [TAPSE] 21 ± 6 mm), and RV-pulmonary artery (PA) coupling index was higher in the non-A2-P2 prolapse/flail patients compared with the A2-P2 prolapse/flail patients (median TAPSE/systolic PA pressure ratio 0.500 [0.341-0.601] vs. 0.383 [0.246-0.442], *p* = 0.038). Relevant (≥moderate) tricuspid regurgitation was prevalent (123/293, 42.0%) without significant differences between groups.

**Table 2:** Baseline echocardiographic characteristics

	All patients N = 315	A2-P2 prolapse/flail N = 129	Non-A2-P2 prolapse/flail N = 186	*p* value
MR severity				0.199
Moderate-to-severe (3+)	49 (15.6)	16 (12.4)	33 (17.7)	
Severe (4+)	266 (84.4)	113 (87.6)	153 (82.3)	
Main pathology causing PMR				**<0.001**
Flail	146 (50.2)	93 (72.1)	53 (28.5)	
Prolapse	102 (35.1)	36 (27.9)	66 (35.5)	
Calcification	7 (2.4)	0	7 (3.8)	
Cleft or indentation	2 (0.7)	0	2 (1.1)	
Restriction	33 (11.3)	0	33 (17.7)	
LVEF (%) (N = 302)	58 ± 10	59 ± 10	58 ± 10	0.357
LVEF <40%	34 (11.3)	15 (11.9)	19 (10.8)	
LVEF 40%-49%	22 (7.3)	6 (4.8)	16 (9.1)	
LVEF ≥50%	246 (81.5)	105 (83.3)	141 (80.1)	
LVEDV (ml)	124 ± 57	122 ± 65	126 ± 54	0.850
LVEDVi (ml/m2)	71 ± 27	70 ± 25	72 ± 30	0.664
LVESV (ml)	114 ± 46	110 ± 45	116 ± 47	0.476
LVEDD (mm)	53 ± 8	52 ± 9	53 ± 8	0.776
LVESD (mm)	39 ± 8	39 ± 10	39 ± 7	0.989
LAVI biplane (ml/m2)	74 ± 30	74 ± 27	74 ± 33	0.993
MVOA (cm2)	5.2 ± 1.8	5.0 ± 2.2	5.3 ± 1.5	0.625
Mean MV pressure gradient (mmHg)	2 [2-3]	2 [2-3]	2 [2-3]	0.796
Max. vena contracta (mm)	7 ± 2	7 ± 2	7 ± 2	0.520
EROA (cm2)	0.4 [0.2 - 0.5]	0.4 [0.2 - 0.8]	0.4 [0.2 - 0.5]	0.154
MR regurgitant volume (ml)	67 ± 48	70 ± 53	64 ± 45	0.656
TAPSE (mm)	21 ± 6	20 ± 5	21 ± 6	0.543
TAPSE∗/SPAP ratio	0.414 [0.296-0.557]	0.383 [0.246-0.442]	0.500 [0.341-0.601]	**0.038**
TR severity (N = 293)				0.761
None	33 (11.3)	16 (13.7)	17 (9.7)	
Mild	137 (46.8)	53 (45.3)	84 (47.7)	
Moderate	82 (28.0)	33 (28.2)	49 (27.8)	
Severe	40 (13.7)	15 (12.8)	25 (14.2)	
Massive	1 (0.3)	0	1 (0.6)	
Torrential	0	0	0	

Results are expressed as absolute number (percentage) for categorical variables and mean (±SD) or median (interquartile range) for continuous variables.

Abbreviations: EROA, effective regurgitant orifice area; LAVI, left atrial volume endex; LVEDD, left ventricular end-diastolic diameter; LVEDV, left ventricular end diastolic volume; LVEDVi, indexed left ventricular end-diastolic volume; LVEF, left ventricular ejection fraction; LVESD, left ventricular end-systolic diameter; LVESV, left ventricular end systolic volume; MR, mitral regurgitation; MV, mitral valve; MVOA, mitral valve orifice area; PMR, primary mitral regurgitation; SPAP, systolic pulmonary artery pressure; TAPSE, tricuspid annular plane systolic excursion; TR, tricuspid regurgitation.

∗ SPAP was measured invasively, and when invasive measurement was unavailable, an echocardiographic estimation was used.

### Procedural and Discharge Outcomes after M-TEER

**Figure 1:**
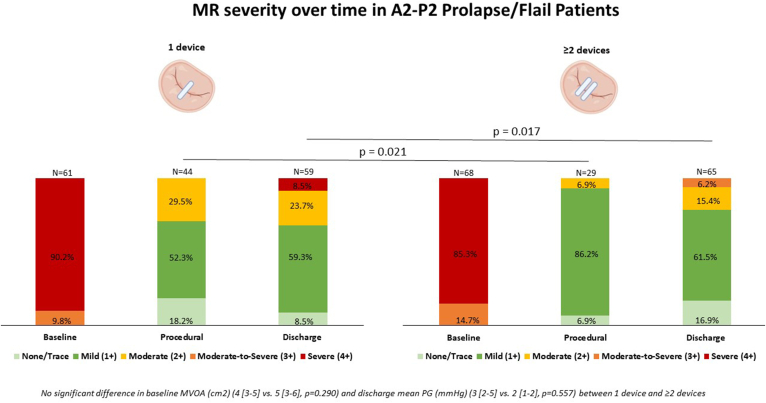
**MR severity over time according to the number of M-TEER devices implanted in A2-P2 prolapse/flail patients.** Abbreviations: MR, mitral regurgitation; M-TEER, mitral transcatheter edge-to-edge repair; MVOA, mitral valve orifice area; PG, pressure gradient.

Procedural and in-hospital outcomes are summarized in Table 3 and Supplementary Table 2, respectively. M-TEER was successful in 93.0% (200/215), without a significant difference between groups according to anatomical lesion. Compared to non-A2-P2 prolapse/flail patients, A2-P2 prolapse/flail received more M-TEER devices (2 [1-2] vs. 1 [1-2], *p* = 0.048) and had more effective MR reduction at discharge (residual MR ≤ 1+: 70.5% [91/125] vs. 60.4% [116/186]; *p* = 0.031) (Graphical Abstract). In patients with A2-P2 prolapse/flail, the implantation of ≥2 M-TEER devices was associated with greater MR reduction at discharge compared with a conservative strategy of 1 M-TEER device, without evidence of MV iatrogenic stenosis and no significant difference in baseline MVA (Figure 1).

**Table 3:** Procedural M-TEER outcomes

	All patients N = 315	A2-P2 prolapse/flail N = 129	Non-A2-P2 prolapse/flail N = 186	*p* value
Number of M-TEER device(s)	1 [1-2]	2 [1-2]	1 [1-2]	**0.048**
1 implant	174 (55.2)	61 (47.3)	113 (60.8)	
2 implants	122 (38.7)	62 (48.1)	60 (32.3)	
3 implants	16 (5.1)	6 (4.7)	10 (5.4)	
4 implants	3 (1.0)	0	3 (1.6)	
Type of M-TEER devices(s)				0.112
MitraClip	250 (79.4)	108 (83.7)	142 (76.3)	
PASCAL	65 (20.6)	21 (16.3)	44 (23.7)	
New iteration of M-TEER devices (MitraClip G4 or PASCAL Ace)	120 (38.1)	42 (32.6)	78 (41.9)	0.092
Residual MR (N = 182, 73 in A2-P2 and 109 in non-A2-P2)				**0.032**
None/trace (0)	14 (7.7)	10 (13.7)	4 (3.7)	
Mild (1+)	122 (67.0)	48 (65.8)	74 (67.9)	
Moderate (2+)	42 (23.1)	15 (20.5)	27 (24.8)	
Moderate-to-severe (3+)	0	0	0	
Severe (4+)	4 (2.2)	0	4 (3.7)	
Technical success (MVARC definition) (N = 215)	200 (93.0)	73 (92.4)	127 (93.4)	0.786
Device embolization	0	0	0	NA
SLDA	1 (0.3)	0	1 (0.5)	0.404
Partial	0		0	
Complete	1 (0.3)		1 (0.5)	
Procedural mortality	0	0	0	NA
Procedural stroke	4 (1.3)	2 (1.6)	2 (1.1)	0.711
Conversion to open MV surgery	0	0	0	NA

Results are expressed as absolute number (percentage) for categorical variables and mean (±SD) or median (interquartile range) for continuous variables.

Abbreviations: MR, mitral regurgitation; M-TEER, mitral transcatheter edge-to-edge repair; MV, mitral valve; MVARC, Mitral Valve Academic Research Consortium; SLDA, single leaflet device attachment.

The rates of in-hospital cerebrovascular events (5/315, 1.6%), major bleedings (1/215, 0.5%), and major vascular complications (6/215, 2.8%) were low without significant differences between groups. All patients were alive at discharge, and none of them underwent MV reintervention.

### Midterm Outcomes After M-TEER

One-year and last follow-up (median of 13 months [5-33], minimum 0 and maximum 72) outcomes are summarized in Table 4 and Supplementary Table 3, respectively. There were no statistically significant differences in baseline characteristics and technical success rate between patients with and without available 1-year clinical follow-up data (Supplementary Table 4), and similar findings were observed when comparing patients with and without available 1-year echocardiographic follow-up.

**Table 4:** One-year outcomes after M-TEER

	All patients N = 202	A2-P2 prolapse/flail N = 85	Non-A2-P2 prolapse/flail N = 117	*p* value
Residual MR severity (N = 168, 73 in A2-P2 and 95 in non-A2-P2)				0.284
None/trace (0)	7 (4.2)	3 (4.1)	4 (4.2)	
Mild (1+)	79 (47.0)	37 (50.7)	42 (44.2)	
Moderate (2+)	59 (35.1)	26 (35.6)	33 (34.7)	
Moderate-to-severe (3+)	8 (4.8)	3 (4.1)	5 (5.3)	
Severe (4+)	15 (8.9)	4 (5.5)	11 (11.6)	
Residual MR ≤ mild (N = 168, 73 in A2-P2 and 95 in non-A2-P2)	86 (51.2)	40 (54.8)	46 (48.4)	0.413
Mean transvalvular MV pressure gradient (mmHg)	4 [3-5]	3 [3-4]	4 [3-5]	0.748
LVEF (%)	56 ± 11	55 ± 11	56 ± 11	0.481
LVEDV (ml)	109 ± 47	120 ± 56	102 ± 40	0.253
LVEDD (mm)	50 ± 7	51 ± 6	49 ± 8	0.122
LAVI biplane (ml/m2)	70 ± 30	69 ± 35	71 ± 27	0.859
TAPSE (mm)	21 ± 6	21 ± 6	20 ± 6	0.408
Estimated SPAP (mmHg)	41 [36-52]	49 [35-53]	39 [35-51]	0.531
TR severity (N = 163, 70 in A2-P2 and 93 in non-A2-P2)				0.513
None/trace	32 (19.6)	15 (21.4)	17 (18.3)	
Mild	70 (42.9)	33 (4.1)	37 (39.8)	
Moderate	47 (28.8)	18 (25.7)	29 (31.2)	
Severe	14 (8.6)	4 (5.7)	10 (10.8)	
Massive	0	0	0	
Torrential	0	0	0	
NYHA functional class (N = 183, 81 in A2-P2 and 102 in non-A2-P2)				**0.017**
I	59 (32.2)	32 (39.5)	27 (26.5)	
II	91 (49.7)	42 (51.9)	49 (48.0)	
III	30 (16.4)	7 (8.6)	23 (22.5)	
IV	3 (1.6)	0	3 (2.9)	
Stroke (N = 215, 76 in A2-P2 and 134 in non-A2-P2)	5 (2.3)	3 (3.8)	2 (1.5)	0.359
TIA (N = 215, 76 in A2-P2 and 134 in non-A2-P2)	1 (0.5)	0	1 (0.7)	0.445
Myocardial infarction (N = 135, 53 in A2-P2 and 82 in non-A2-P2)	1 (0.7)	1 (1.9)	0	0.212
Bleeding complications (N = 134, 52 in A2-P2 and 82 in non-A2-P2)	3 (2.2)	0	3 (3.7)	0.378
None	131 (97.8)		79 (96.3)	
Minor	2 (1.5)		2 (2.4)	
Major	1 (0.7)		1 (1.2)	
Life-threatening	0		0	
Fatal	0		0	
HFH (N = 136, 52 in A2-P2 and 84 in non-A2-P2)	8 (5.9)	2 (3.8)	6 (7.1)	0.427
Reason for HFH (N = 136, 52 in A2P2 and 84 in non-A2P2)				
Recurrence of ≥ grade 3 MR	1 (0.7)		1 (1.2)	
Partial SLDA (partial detachment of the posterior leaflet) and severe atrial SMR	1 (0.7)	1 (1.9)		
Recurrence of A2-flail	2 (1.5)		2 (2.4)	
Recurrence of posterior prolapse due to Barlow	1 (0.7)	1 (1.9)		
Iatrogenic MV stenosis	1 (0.7)		1 (1.2)	
Iatrogenic ASD with left right-shunt and right-heart volume overload after M-TEER				
Severe secondary TR	2 (1.5)		2 (2.4)	
SLDA (N = 165, 73 in A2-P2 and 92 in non-A2-P2)	5 (3.0)		3 (3.3)	0.965
Partial	1 (0.6)	2 (2.7)	1 (1.1)	
Complete	4 (2.4)	2 (2.7)	2 (2.2)	
MV reintervention (N = 202, 85 in A2-P2 and 117 in non-A2-P2)			0	0.367
Type of MV reintervention	1 (0.3)	1 (0.7)		
Elasta-Clip followed by transapical TMVI	1 (0.3)	1 (0.7)		
All-cause mortality (N = 203, 86 in A2-P2 and 117 in non-A2-P2)	35 (17.2)	13 (15.1)	22 (18.8)	0.492

Results are expressed as absolute number (percentage) for categorical variables and mean ±SD or median [interquartile range] for continuous variables.

Abbreviations: ASD, atrial septal defect; HFH, heart failure rehospitalization; LAVI, left atrial volume endex; LVEDD, left ventricular end-diastolic diameter; LVEDV, left ventricular end diastolic volume; LVEF, left ventricular ejection fraction; MR, mitral regurgitation; M-TEER, mitral transcatheter edge-to-edge repair; MV, mitral valve; NYHA, New York Heart Association; SLDA, single leaflet device attachment; SMR, secondary mitral regurgitation; SPAP, systolic pulmonary artery pressure; TAPSE, tricuspid annular plane systolic excursion; TIA, transient ischemic attack; TMVI, transcatheter mitral valve implantation; TR, tricuspid regurgitation.

**Figure 2:**
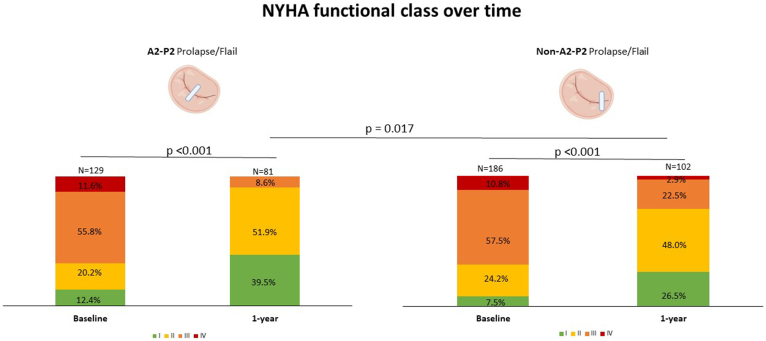
**NYHA functional class over time according to the characteristics of the lesion causing PMR.** Abbreviations: NYHA, New York Heart Association; PMR, primary mitral regurgitation.

MR was significantly reduced at 1 year (MR ≤ 2+ in 86.3%, 145/168), without a significant difference according to the lesion causing PMR (*p* = 0.284) (Graphical Abstract). This resulted in significant NYHA functional class improvement at 1 year in the overall cohort (class ≤ II in 82%, 150/183), with A2-P2 prolapse/flail patients showing superior symptomatic improvement at 1 year compared with the non-A2-P2 prolapse/flail patients (Figure 2). The rates of stroke (5/215, 2.3%) and HF hospitalization (HFH) (8/126, 5.9%) remained low at 1 year and were similar between groups. The reasons for HFH are specified in Table 4. At 1 year, five patients (5/165, 2.7%) were found to have partial or complete SLDA, which was not associated with the lesion causing PMR or the number of devices implanted. One patient underwent MV reintervention, consisting of electrosurgical anterior leaflet laceration and stabilization of the implant (ELASTA-Clip) followed by transcatheter mitral valve implantation.^27^ One-year all-cause mortality was 17.2% (35/203) and did not differ between A2-P2 prolapse/flail and non-A2-P2 prolapse/flail groups (the corresponding Kaplan-Meier curve is shown in Supplementary Figure 1).

An analysis based on the type of device used (newer iterations of M-TEER devices [MitraClip G4 or PASCAL Ace] vs. earlier generations) revealed no significant difference in achieving residual MR ≤ 1+ at last follow-up within the overall cohort (45.6% [41/90] vs. 44.4% [48/108], *p* = 0.876). Similar findings were observed in both the A2-P2 prolapse/flail group and the non-A2-P2 prolapse/flail group.

In patients with A2-P2 prolapse/flail, although the proportion of patients with residual MR ≤ 1+ at 1 year was higher in the ≥2 M-TEER devices group than in the single M-TEER device group (59.6% [28/47] vs. 46.2% [12/26]), this difference was not statistically significant (*p* = 0.330). Likewise, the overall distribution of MR grades did not differ significantly between groups (*p* = 0.148). A similar trend toward lower mortality was observed in the ≥2 M-TEER devices group compared with the single M-TEER device group at 1 year (10.0% [5/50] vs. 22.2% [8/36]), although this difference was not statistically significant (*p* = 0.119).

In the subgroup of patients with preinterventional TEE 3D images, 1-year all-cause mortality was higher among those with complex MV anatomy compared with noncomplex anatomy (23.2% [13/56] vs. 8.2% [5/61], *p* = 0.038). At the last follow-up (median 22 months [9–36], minimum 0 and maximum 72), this difference remained significant (51.5% [35/68] vs. 34.7% [26/75], *p* = 0.042) (Supplementary Table 6). Although not statistically significant, there was a trend toward a higher proportion of patients with residual MR ≤ mild in the noncomplex MV anatomy group compared to the complex MV anatomy group (46.9 vs. 34.5%) (Supplementary Table 6). This difference in outcomes occurred despite the complex MV anatomy group being younger (79.4 ± 7.0 years vs. 82.7 ± 5.9 years; *p* = 0.003) and having a similar or lower baseline comorbidity burden, including better renal function (Supplementary Table 5).

### Predictors of One-Year Mortality

Compared with survivors, deceased patients at 1 year had more frequent severe renal failure, tended to have lower RV-PA coupling ratio (TAPSE/PASP), were more likely to have complex MV anatomy, and had higher serum creatinine and n-terminal pro-B type natriuretic peptide levels (Table 5). Using multivariate analysis, severe renal failure (HR: 19.80; 95% CI: 2.68-146.04; *p* = 0.003) and complex MV anatomy (HR: 10.01; 95% CI: 1.30-77.04; *p* = 0.027) were identified as independent predictors of 1-year all-cause mortality (Table 6). The corresponding Kaplan-Meier curve according to anatomical MV complexity is shown in the Graphical Abstract.

**Table 5:** Characteristics of surviving and deceased patients at 1 year after M-TEER

	Alive N = 168	Deceased N = 35	*p* value
Age at M-TEER (y)	81.8 ± 6.8	81.7 ± 7.6	0.949
Female	75 (44.6)	19 (54.3)	0.298
EuroSCORE II (%)	4.3 ± 2.8	7.6 ± 7.1	**0.002**
STS score for MV replacement (%)	3.5 ± 2.7	6.3 ± 6.2	**0.002**
BMI (kg/m2)	24.2 ± 4.2	24.2 ± 4.2	0.980
Obesity (BMI ≥30) (N = 202, 168 alive and 35 deceased)	17 (10.1)	3 (8.6)	0.780
Arterial hypertension (N = 202, 168 alive and 35 deceased)	122 (73.1)	30 (85.7)	0.115
Severe renal failure (eGFR <30 ml/min/1.73 m2)	29 (17.3)	13 (37.1)	**0.012**
Diabetes	17 (10.1)	2 (5.7)	0.328
Dyslipidemia	65 (38.7)	13 (37.1)	0.864
History of malignancy (N = 186, 152 alive and 34 deceased)	34 (22.4)	12 (35.3)	0.127
Atrial fibrillation	88 (52.4)	19 (54.3)	0.855
Chronic obstructive pulmonary disease	14 (8.3)	6 (17.1)	0.123
Coronary artery disease	55 (32.7)	15 (42.9)	0.328
Prior myocardial infarction	14 (8.3)	3 (8.6)	0.963
Prior percutaneous coronary intervention	43 (25.6)	11 (31.4)	0.529
History of stroke	15 (8.9)	5 (14.3)	0.351
Pulmonary hypertension (mPAP ≥20 mmHg) (N = 72, 62 alive and 10 deceased)	51 (82.3)	7 (70.0)	0.397
Anemia (female: Hb < 120 g/L; male: Hb < 130 g/L)	77 (45.8)	20 (57.1)	0.266
Previous TAVI (N = 134, 115 alive and 19 deceased)	5 (4.3)	1 (5.3)	0.858
Previous SAVR (N = 134, 115 alive and 19 deceased)	6 (5.2)	1 (5.3)	0.993
Previous surgical MV repair	4 (2.4)	0	0.357
Previous CABG	12 (7.1)	6 (17.1)	0.094
HFH in the last 12 mo prior to M-TEER (N = 117, 99 alive and 18 deceased)	30 (30.3)	6 (33.3)	0.787
NYHA functional class III or IV	108 (64.3)	25 (71.4)	0.419
eGFR (ml/min)	47 [36-66]	48 [28-65]	0.230
Creatinine (μmol/L)	95 [78-126]	100 [91-161]	**0.036**
NT-proBNP (pg/ml)	2145 [828 - 4960]	3951 [1941-7672]	**0.032**
MR severity			0.667
Moderate-to-severe (3+)	33 (19.6)	8 (22.9)	
Severe (4+)	135 (80.4)	27 (77.1)	
A2-P2 prolapse/flail causing PMR	73 (43.5)	13 (37.1)	0.574
Anatomically∗ complex MV (N = 117, 99 alive and 18 deceased)	43 (43.4)	13 (72.2)	**0.025**
≥Moderate MV calcifications (N = 117, 99 alive and 18 deceased)	5 (5.1)	3 (16.7)	0.072
Barlow’s disease (N = 117, 99 alive and 18 deceased)	13 (13.1)	6 (33.3)	0.074
Multiple MV prolapses or commissural MV prolapse (N = 117, 99 alive and 18 deceased)	37 (37.4)	10 (55.6)	0.148
LVEF (%)	58 ± 11	57 ± 10	0.476
LVEF <40%	21 (12.8)	6 (17.1)	0.310
LVEF 40%-49%	9 (5.5)	4 (11.4)	
LVEF ≥50%	134 (81.7)	25 (71.4)	
LVEDV (ml)	132 ± 66	121 ± 22	0.712
LVEDD (mm)	52 ± 8	53 ± 9	0.764
MVOA (cm2)	5.0 ± 1.9	5.1 ± 1.9	0.856
Mean MV pressure gradient (mmHg)	2 [2-3]	2 [2-3]	0.519
Max. vena contracta (mm)	7 ± 2	7 ± 1	0.959
TAPSE (mm)	21 ± 6	17 ± 7	0.052
TAPSE†/SPAP ratio	0.463 [0.284-0.596]	0.309 [0.203-0.383]	0.055
TR severity (N = 188, 155 alive and 33 deceased)			0.136
None	19 (12.3)	3 (9.1)	
Mild	73 (47.1)	12 (36.4)	
Moderate	43 (27.7)	10 (30.3)	
Severe	20 (12.9)	7 (21.2)	
Massive	0	1 (3.0)	
Torrential	0	0	

Results are expressed as absolute number (percentage) for categorical variables and mean (±SD) or median (interquartile range) for continuous variables.

Abbreviations: BMI, body mass index; CABG, coronary artery bypass graft; eGFR, estimated glomerular filtration rate; EuroSCORE II, European System for Cardiac Operative Risk Evaluation II; Hb, hemoglobin; HFH, heart failure rerehospitalization; LVEDD, left ventricular end-diastolic diameter; LVEDV, left ventricular end diastolic volume; LVEF, left ventricular ejection fraction; mPAP, mean pulmonary artery pressure; MR, mitral regurgitation; M-TEER, mitral transcatheter edge-to-edge repair; MV, mitral valve; MVOA, mitral valve orifice area; NT-proBNP, N-terminal pro–B-type natriuretic peptide; NYHA, New York Heart Association; PMR, primary mitral regurgitation; SAVR, surgical aortic valve replacement; SPAP, systolic pulmonary artery pressure; STS, Society of Thoracic Surgeons; TAPSE, tricuspid annular plane systolic excursion; TAVI, transcatheter aortic valve implantation; TR, tricuspid regurgitation.

∗ Anatomical complexity defined as presence of ≥1 of the following criteria: ≥moderate calcifications, Barlow’s disease, multiple prolapses, or commissural prolapse.

† SPAP was measured invasively, and when invasive measurement was unavailable, an echocardiographic estimation was used.

**Table 6:** Predictors of 1-year all-cause mortality after M-TEER (multivariate analysis)

	All-cause mortality HR (95% CI)	*p* value
Severe renal failure (eGFR <30 ml/min/1.73 m2)	19.80 (2.68 – 146.04)	**0.003**
Coronary artery disease	3.75 (0.68 – 50.6)	0.111
Complex MV anatomy∗	10.01 (1.30 – 77.04)	**0.027**
LV systolic dysfunction (LVEF<50%)	1.14 (0.12 – 10.7)	0.912
RV-PA uncoupling†	1.50 (0.31 – 7.4)	0.617

Cox regression (proportional hazards regression) for mortality analysis at 1-year after M-TEER.

Note. Similar results were found when age was integrated into the model (age not associated with 1-year all-cause mortality; wider CIs for severe renal failure and complex MV anatomy).

Abbreviations: eGFR, estimated glomerular filtration rate; HR, hazard ratio; LV, left ventricular; LVEF, left ventricular ejection fraction; M-TEER, mitral transcatheter edge-to-edge repair; MV, mitral valve; PA, pulmonary artery; RV, right ventricular; SPAP, systolic pulmonary artery pressure; TAPSE, tricuspid annular plane systolic excursion.

∗ Anatomical complexity defined as presence of ≥1 of the following criteria: ≥moderate calcifications, Barlow’s disease, multiple prolapses, or commissural prolapse.

† RV-PA uncoupling defined as TAPSE/SPAP <0.307.^28^ SPAP was measured invasively, and when invasive measurement was unavailable, an echocardiographic estimation was used.

## Discussion

In the present study, we evaluated the short-term and midterm outcomes of M-TEER according to PMR etiology and anatomical characteristics. The main findings are as follows:

1) Demonstrating a high technical success rate (>90%), M-TEER effectively reduced PMR and alleviated symptoms irrespective of the underlying MR mechanism.
2) Patients with A2-P2 prolapse/flail exhibited superior short-term echocardiographic outcomes and greater symptomatic improvement at 1 year compared with those with non-A2–P2 prolapse/flail lesions.
3) Among patients with A2-P2 prolapse/flail, implantation of two or more devices achieved greater MR reduction at discharge than single-device implantation.
4) While clinical comorbidities are recognized predictors of mortality, our findings highlight complex MV anatomy as an additional independent predictor of 1-year all-cause mortality after M-TEER.

Our study investigating M-TEER for PMR according to anatomy is the first to include patients treated over a period of more than 10 years. Our results confirm the safety of M-TEER in PMR patients at high surgical risk, including those with less favorable anatomies. Compared to the MitraClip EXPAND G4 study,^10^ our cohort exhibited similar rates of stroke (∼2%) and complete SLDA (∼2%), a lower rate of HFH (5.9 vs. ∼ 10%), and only 1 MV reintervention at 1 year. Our observed all-cause mortality rate (17.2%) aligns with recent real-world data from the Society of Thoracic Surgeons/American College of Cardiology Transcatheter Valve Therapy Registry (15.4%).^11^

Effectiveness analysis showed that patients with central and simple lesions (A2-P2 prolapse/flail) had a more effective reduction in MR at discharge (residual MR ≤ 1+: 70.5 vs. 60.4%; *p* = 0.031) compared to non-A2-P2 prolapse/flail. Importantly, in patients with PMR, achieving residual MR ≤ 1+ is of prognostic relevance.^11^ Previous studies have indicated higher rates of recurrent symptomatic MR and MV reintervention in PMR patients with complex anatomies, such as flail width >15 mm, flail gap ≥10 mm,^14^ and Barlow’s disease.^29^ Furthermore, anatomical features like annular calcification with or without leaflet involvement or calcified landing zone, MVA ≤ 4cm2, baseline transmitral gradient ≥4 mm Hg, and multiple jets have been associated with increased final transmitral gradient (≥5 mm Hg) after M-TEER.^30^ Over the years, advancements in device technology and accumulated expertise of multidisciplinary teams in high-volume valve centers have enabled the safe and effective treatment of PMR patients previously deemed anatomically complex and unsuitable for M-TEER.^31^^,^^32^ The emergence of dedicated transcatheter MV bioprostheses^33^ and improved prediction methods of the risk of left ventricular outflow tract obstruction (fixed and dynamic),^34^^,^^35^ as well as the advent of new transcatheter strategies to prevent left ventricular outflow tract obstruction,^36^ offer an alternative for nonoperable patients who are not suitable for M-TEER. Recently, a group of experts proposed criteria for choosing between M-TEER and MV replacement (surgical or transcatheter) based on anatomical complexity and center experience.^22^

In addition to therapeutic approach and device selection, our study highlights the importance of the number of M-TEER devices implanted in patients with presumed nonextensive central disease and simple anatomy. Among patients included in the A2-P2 prolapse/flail group, our results suggest that a multidevice implantation strategy effectively reduces MR in the short term without significant risk of iatrogenic MV stenosis. Although limited by loss to follow-up and warranting confirmation in larger prospective studies, 1-year results were consistent with short-term findings, suggesting a nonsignificant trend toward better MR reduction and survival associated with a ≥2 M-TEER device strategy compared with a single device in patients with simple central MV lesions. Our findings are consistent with and build upon a previous single-center retrospective study,^37^ which demonstrated that freedom from MR ≥ 3+ 2 years following M-TEER in PMR patients was significantly greater among those treated with two devices compared to those treated with a single device. Notably, despite a reduction in MVA, this approach was not associated with an increase in all-cause mortality at 2-year follow-up.^37^ An echocardiographic study based on 3D analysis of TEE imaging showed that the minimum native MVA required to avoid clinically relevant MV stenosis after M-TEER depends on the number and location of devices, orifice morphology, and device type.^38^ A predictive algorithm has been developed to optimize patient selection and procedural planning based on these parameters.

A key finding of our study is the independent association of complex MV anatomy, alongside severe renal failure, with 1-year all-cause mortality following M-TEER. This aligns with a recent retrospective study by Sorajja et al. (n = 386 patients; median age 82 years [75–86], 79.3% PMR), which showed reduced technical success and increased 1-year all-cause mortality in patients classified as anatomically unsuitable for M-TEER.^13^ These findings suggest that higher 1-year all-cause mortality in patients with complex MV anatomy undergoing M-TEER may stem from suboptimal procedural outcomes, including less effective MR reduction. Alternatively, this association may be attributed to a higher baseline comorbidity burden in patients with complex anatomy (e.g., severe MV calcifications), reflecting more advanced disease. This latter hypothesis was not supported in our cohort.

Consistent with prior studies, our results confirm that M-TEER leads to significant improvements in functional status—as measured by NYHA class—in patients with PMR regardless of MV anatomy.^31^^,^^32^ However, the magnitude of symptomatic improvement is greater in those with nonextensive central lesions and simpler anatomy. Given that M-TEER is primarily offered to PMR patients who are not candidates for surgery, alleviating symptoms remains a central therapeutic objective.

The expansion of M-TEER to patients with more complex anatomies and potentially longer life expectancy^39^ underscores the critical importance of optimal patient and device selection at the time of the initial intervention for effective lifetime management.^39^^,^^40^ Preprocedural assessment of anatomical complexity plays a key role in evaluating the feasibility of M-TEER, complementing existing anatomical classification systems^22^ through an integrated approach that incorporates the specific morphological features identified on 3D echocardiography. This assessment also enables early referral of patients with challenging anatomies to multidisciplinary Heart Teams at experienced Heart Valve Centers^41^ and supports the selection of an optimal interventional strategy, including the use of multiple M-TEER devices when appropriate. When the anatomy is particularly complex, transcatheter mitral valve implantation should be considered and discussed within the multidisciplinary Heart Team at the Heart Valve Center. The encouraging results of the recent prospective, international, single-arm ENCIRCLE (sapiEN M3 system transCatheter mItral valve ReplaCement via transseptaL accEss) trial^33^ open new perspectives and highlight the importance of assessing additional therapeutic options for nonoperable patients with MV anatomies unsuitable for M-TEER. Furthermore, patients with suboptimal outcomes after M-TEER require specific diagnostic evaluation and may benefit from appropriate transcatheter reintervention.^27^^,^^42^

With ongoing advancements in technology and increased multidisciplinary expertise, M-TEER has matured into the treatment of choice for symptomatic patients with severe secondary MR and is emerging as a competitive alternative to surgery in high-risk PMR patients. This hypothesis is being evaluated in ongoing trials such as REPAIR-MR (Percutaneous MitraClip Device or Surgical Mitral Valve REpair in PAtients With PrImaRy MItral Regurgitation Who Are Candidates for Surgery)^43^ and PRIMARY (Percutaneous or Surgical Repair In Mitral Prolapse And Regurgitation for ≥60 Year-olds) (NCT05051033).

### Study Limitations

This study has inherent limitations related to its partially retrospective and observational design, and its inclusion of solely patients treated at two high-volume centers may introduce selection bias. The absence of independent event adjudication further limits the generalizability of the findings. The modest sample size and loss to follow-up at 1 year also limit interpretation and highlight the need for larger, long-term studies with systematic echocardiographic assessment and finer anatomical granularity to better delineate the individual prognostic contribution of each component of complex MV anatomy. Finally, the study spans a 10-year period, during which substantial advances occurred in device technology, operator experience, and procedural management. These temporal improvements, together with the progressive expansion of M-TEER to older and sicker patients with increasingly complex mitral anatomies, have likely influenced procedural success and midterm outcomes. Although outcomes were compared across device generations, the analysis did not account for calendar time, operator learning curves, or center-specific evolution. Moreover, a more granular assessment of device characteristics—considering specific models and technical refinements rather than broad generational categories—would allow a more precise evaluation of device-related effects. Future studies should use longitudinal or time-dependent modeling approaches, such as mixed-effects or multilevel regression analyses incorporating operator- and center-level experience, as well as temporal covariates, to clarify how each factor influences M-TEER outcomes.

## Conclusions

M-TEER is a safe treatment option for PMR patients at high surgical risk. Nevertheless, preprocedural evaluation should take into account not only clinical comorbidities but also anatomical characteristics that are strongly related to outcomes in PMR patients undergoing M-TEER. Our findings expand on the limited existing data and have significant implications for the selection of PMR patients assessed for M-TEER.

## Funding

The authors have no funding to report.

## Disclosure Statement

D. Samim received institutional funding for an online course from 10.13039/100006520Edwards Lifesciences with no personal payments. J. Bartkowiak reports a research grant from 10.13039/100008273Novartis Foundation. T. Pilgrim reports research grants from the 10.13039/501100001711Swiss National Science Foundation, the 10.13039/501100004362Swiss Heart Foundation, the 10.13039/501100015594Swiss Polar Institute, the Bangerter-Rhyner Foundation, the 10.13039/100011606Mach-Gaensslen Foundation, and the Monsol Foundation; research, travel, or educational grants to the institution without personal remuneration from Biotronik, Boston Scientific, Edwards Lifesciences, and ATSens; speaker fees and consultancy fees to the institution from 10.13039/501100005035Biotronik, 10.13039/100008497Boston Scientific, 10.13039/100006520Edwards Lifesciences, 10.13039/100000046Abbott, 10.13039/100004374Medtronic, Biosensors, and Highlife. P. Biaggi reports proctoring/consulting fees for 10.13039/100000046Abbott. F. Praz was compensated for travel expenses from 10.13039/100011949Abbott Vascular, 10.13039/100006520Edwards Lifesciences, Medira, 10.13039/100015696Siemens Healthineers, and InQB8 Medical Technologies; and has received a research grant to the institution from 10.13039/100011949Abbott Vascular. P.M. Wenaweser reports proctoring/consulting fees from 10.13039/100006520Edwards Lifesciences and 10.13039/100004374Medtronic and honoraria from Daiichi Sankyo. The other authors had no conflicts to declare.
