# Supplementary material for: Midterm Outcomes of Transcatheter Edge-to-Edge Repair for Primary Mitral Regurgitation According to Anatomical Characteristics
Source: Struct Heart. 2025 Nov 17;10(3):100763. doi: 10.1016/j.shj.2025.100763 (PMC12878661; doi:10.1016/j.shj.2025.100763)
Supplement: Supplementary Figure 1 and Tables 1-6 [file mmc1.docx]

**Supplementary Figure 1**: Estimated cumulative survival (Kaplan–Meier curve) of patients with A2-P2 Prolapse/Flail vs. Non-A2-P2 Prolapse/Flail up to one-year follow-up after M-TEER

**
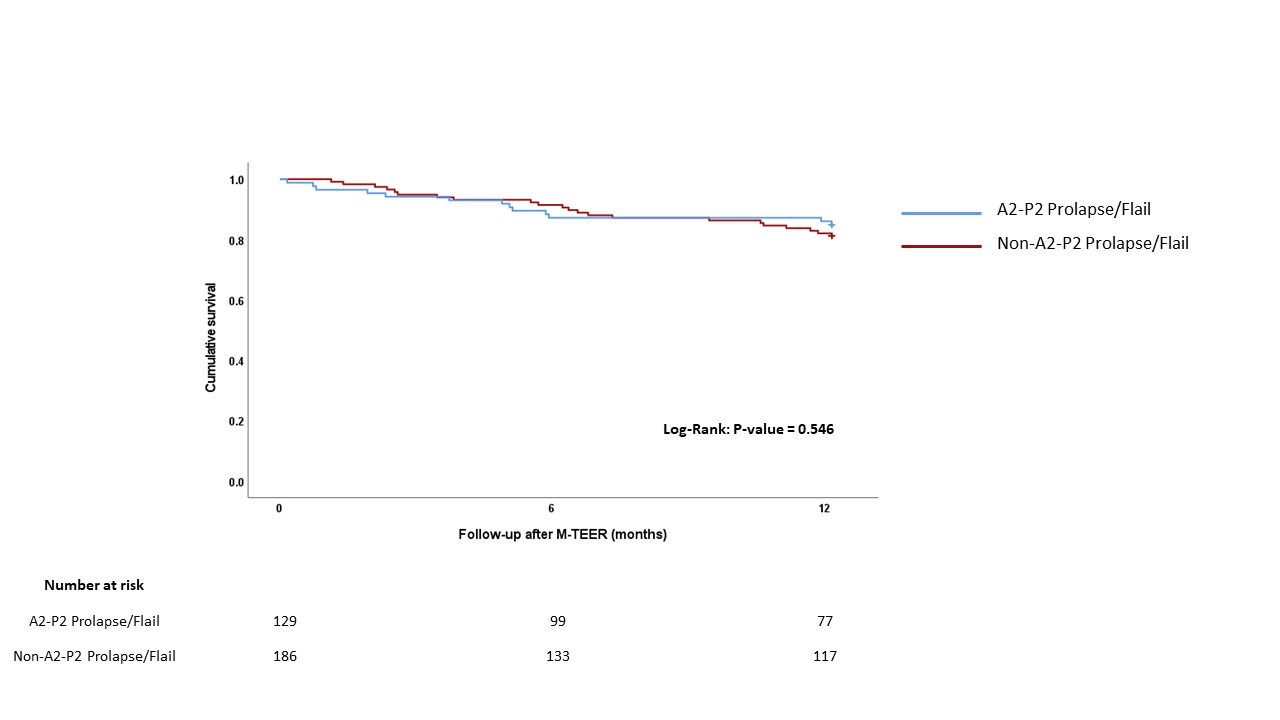
**

**Supplementary Table 1**: baseline invasive hemodynamic measurements

|  | **All patients**  N = 146 | **A2-P2 Prolapse/Flail**  N=59 | **Non-A2-P2 Prolapse/Flail**  N=87 | **P-value** |
| --- | --- | --- | --- | --- |
| Aortic systolic Pressure (mmHg) | 125± 25 | 120± 24 | 128± 27 | 0.064 |
| Aortic diastolic pressure (mmHg) | 64 ± 15 | 62 ± 13 | 65 ± 15 | 0.073 |
| Aortic mean pressure (mmHg) | 88 ± 18 | 85 ± 15 | 90 ± 19 | 0.066 |
| PAP systolic (mmHg) | 46 ± 15 | 49 ± 16 | 44± 15 | **0.035** |
| PAP diastolic (mmHg) | 17 ± 8 | 18 ± 7 | 17 ± 8 | 0.380 |
| PAP mean (mmHg) | 30 ± 11 | 32 ± 10 | 29 ± 11 | 0.138 |
| Pulmonary Hypertension (mPAP ≥ 20 mmHg)  (N=133, 51 in A2-P2 and 82 in Non-A2-P2) | 106 (33.7) | 44 (86.3) | 62 (75.6) | 0.137 |
| LV systolic pressure (mmHg) | 128 ± 27 | 126 ± 26 | 129 ± 29 | 0.600 |
| LVEDP (mmHg) | 16 ± 7 | 16 ± 6 | 16 ± 7 | 0.761 |
| RA mean pressure (mmHg) | 8 ± 5 | 8 ± 4 | 8 ± 5 | 0.793 |
| PCWP mean (mmHg) | 19 ± 8 | 20 ± 9 | 18 ± 8 | 0.397 |

Results are expressed as absolute number (percentage) for categorical variables and mean (±SD) or median [interquartile range] for continuous variables.

LV = left ventricular; LVEDP = left ventricular end-diastolic pressure; mPAP = mean pulmonary artery pressure; PAP = pulmonary artery pressure; PCWP; pulmonary capillary wedge pressure; RA = right atrial

**Supplementary Table 2**: In-hospital outcomes after M-TEER

|  | **All patients**  N = 315 | **A2-P2 Prolapse/Flail**  N=129 | **Non-A2-P2 Prolapse/Flail**  N=186 | **P-value** |
| --- | --- | --- | --- | --- |
| Residual MR severity  (N=306, 124 in A2-P2 and 182 in Non-A2-P2)  None/trace (0)  Mild (1+)  Moderate (2+)  Moderate-to-severe (3+)  Severe (4+) | 28 (9.2)  179 (58.5)  74 (24.2)  8 (2.6)  17 (5.6) | 16 (12.9)  75 (60.5)  24 (19.4)  4 (3.2)  5 (4.0) | 12 (6.6)  104 (57.1)  50 (27.5)  4 (2.2)  12 (6.6) | **0.031** |
| Mean transvalvular MV pressure gradient (mmHg) | 3 [3-5] | 3 [3-4] | 4 [3-5] | 0.247 |
| Estimated SPAP (mmHg) | 42 [35-52] | 39 [34-50] | 45 [38-53] | 0.117 |
| TR severity (N=303, 122 in A2-P2 and 181 in Non-A2-P2))  None/trace  Mild  Moderate  Severe  Massive  Torrential | 47 (15.5)  157 (51.8)  66 (21.8)  33 (10.9)  0  0 | 21 (17.2)  68 (55.7)  25 (20.5)  8 (6.6)  0  0 | 26 (14.4)  89 (49.2)  41 (22.7)  25 (13.8)  0  0 | 0.198 |
| Haemoglobin value (g/l) | 110 [100-123] | 113 [101-123] | 110 [99-123] | 0.353 |
| NTproBNP value (pg/ml) | 1’128 [179-2’602] | 924 [222-2’698] | 1’187 [224-2’473] | 0.960 |
| Creatinine (umol/l) | 125 [99-136] | 89 [74-121] | 90 [74-120] | 0.670 |
| SLDA  Partial  Complete | 1 (0.3)  0  1 (0.3) | 0 | 1 (0.5)  1 (0.5)  0 | 0.404 |
| Stroke | 5 (1.6) | 3 (2.3) | 2 (1.1) | 0.359 |
| TIA | 0 | 0 | 0 | NA |
| Myocardial infarction (N=215, 79 in A2-P2 and 136 in Non-A2-P2) | 1 (0.5) | 1 (1.3) | 0 | 0.188 |
| Bleeding complications (N=215, 79 in A2-P2 and 136 in Non-A2-P2)  None  Minor  Major  Life-threatening  Fatal | 2 (0.9)  213 (99.0)  1 (0.5)  1 (0.5)  0  0 | 0 | 2 (1.5)  134 (98.5)  1 (0.7)  1 (0.7)  0  0 | 0.556 |
| Vascular complications (N=215, 79 in A2-P2 and 136 in Non-A2-P2)  None  Minor access site vascular complication  Major access site vascular complication | 6 (2.8)  209 (97.2)  0  6 (2.8) | 2 (2.5)  77 (97.5)  0  2 (2.5) | 4 (2.9)  132 (97.1)  0  4 (2.9) | 0.860 |
| MV re-intervention | 0 | 0 | 0 | NA |
| All-cause mortality | 0 | 0 | 0 | NA |

Results are expressed as absolute number (percentage) for categorical variables and mean (±SD) or median [interquartile range] for continuous variables. If not specified, N (denominator) = 315

M-TEER = mitral transcatheter edge-to-edge repair; MR = mitral regurgitation; MV = mitral valve; SLDA = single leaflet device attachment; SPAP = systolic pulmonary artery pressure; SMR = secondary mitral regurgitation; TAPSE = tricuspid annular plane systolic excursion; TIA = transient ischemic attack: TMVI = transcatheter mitral valve implantation; TR = tricuspid regurgitation

**Supplementary Table 3**: Last follow-up (median of 13 months [5-33], minimum 0 and maximum 72) outcomes after M-TEER

|  | **All patients**  N=315 | **A2-P2 Prolapse/Flail**  N=129 | **Non-A2-P2 Prolapse/Flail**  N=186 | **P-value** |
| --- | --- | --- | --- | --- |
| Residual MR severity (N=198, 71 in A2-P2 and 127 in Non-A2-P2)  None/trace (0)  Mild (1+)  Moderate (2+)  Moderate-to-severe (3+)  Severe (4+) | 15 (7.6)  76 (38.4)  68 (34.3)  19 (9.6)  20 (10.1) | 4 (5.6)  31 (43.7)  27 (38.0)  4 (5.6)  5 (7.0) | 11 (8.7)  45 (35.4)  41 (32.3)  15 (11.8)  15 (11.8) | 0.317 |
| Residual MR ≤ Mild | 89 (44.9) | 34 (47.9) | 55 (43.3) | 0.534 |
| Mean transvalvular MV pressure gradient (mmHg) | 4 [3-5] | 4 [3-4] | 4 [3-5] | 0.150 |
| LVEF (%) | 55 ± 11 | 55 ± 11 | 55 ± 11 | 0.781 |
| LVEDV (ml) | 116 ± 53 | 113 ± 41 | 118 ± 59 | 0.741 |
| LVEDD (mm) | 50 ± 8 | 51 ± 8 | 50 ± 8 | 0.549 |
| LAVI biplane (ml/m2) | 63 ± 44 | 57 ± 42 | 67 ± 45 | 0.337 |
| TAPSE (mm) | 19 ± 6 | 20 ± 6 | 18 ± 6 | 0.065 |
| Estimated SPAP (mmHg) | 43 [34-51] | 45 [37-55] | 42 [33-50] | 0.154 |
| TR severity (N=180, 65 in A2-P2 and 115 in Non-A2-P2)  None/trace  Mild  Moderate  Severe  Massive  Torrential | 26 (14.4)  74 (41.1)  54 (30.0)  26 (14.1)  0  0 | 11 (16.9)  27 (41.5)  19 (29.2)  8 (12.3)  0  0 | 15 (13.0)  47 (40.9)  35 (30.4)  18 (15.7)  0  0 | 0.855 |
| NYHA functional class  (N=194, 78 in A2-P2 and 116 in Non-A2-P2)  I  II  III  IV | 43 (22.2)  105 (54.1)  41 (21.1)  5 (2.6) | 23 (29.5)  45 (57.7)  9 (11.5)  1 (1.3) | 20 (17.2)  60 (51.7)  32 (27.6)  4 (3.4) | **0.019** |
| Stroke (N=215, 76 in A2-P2 and 134 in Non-A2-P2) | 7 (3.3) | 3 (3.8) | 4 (2.9) | 0.733 |
| TIA (N=215, 76 in A2-P2 and 134 in Non-A2-P2) | 2 (0.9) | 0 | 2 (1.5) | 0.533 |
| Myocardial infarction (N=215, 79 in A2-P2 and 136 in Non-A2-P2) | 1 (0.5) | 1 (1.3) | 0 | 0.188 |
| Bleeding complications  None  Minor  Major  Life-threatening  Fatal | 3 (1.4)  212 (98.6)  2 (0.9)  1 (0.5)  0  0 | 0 | 3 (2.2)  2 (1.5)  1 (0.7)  0  0 | 0.413 |
| HFH  (N=215, 79 in A2-P2 and 136 in Non-A2-P2) | 9 (3.7) | 3 (3.8) | 6 (4.4) | 0.828 |
| Reason for HFH (N=215, 79 in A2-P2 and 136 in non-A2-P2)  Recurrence of ≥ grade 3 MR  - Partial SLDA (partial detachment of the posterior leaflet) and severe atrial SMR  - Recurrence of A2-Flail  - Recurrence of posterior Prolapse due to Barlow  Iatrogenic MV stenosis  Iatrogenic ASD with left right-shunt and right-heart volume overload after M-TEER  Severe secondary TR | 1 (0.5)  2 (0.9)  2 (0.9)  1 (0.5)  1 0.5)  2 (0.9) | 2 (2.5)  1 (1.2) | 1 (0.7)  2 (1.5)  1 (0.7)  2 (1.5) |  |
| SLDA (N=198, 71 in A2-P2 and 127 in Non-A2-P2)  Partial  Complete | 5 (2.5)  1 (0.5)  4 (2.0) | 2 (2.8)  2 (2.8) | 3 (2.4)  1 (0.8)  2 (1.6) | 0.845 |
| MV re-intervention (N=315, 129 in A2-P2 and 186 in Non-A2-P2)  Type of MV re-intervention  Elasta-Clip followed by transapical TMVI | 3 (0.9)  3 (0.9) | 3 (2.3)  3 (2.3) | 0 | **0.037** |
| All-cause mortality (N=315, 129 in A2-P2 and 186 in Non-A2-P2) | 84 (26.7) | 28 (21.7) | 56 (30.1) | 0.097 |

Results are expressed as absolute number (percentage) for categorical variables and mean (±SD) or median [interquartile range] for continuous variables.

ASD = atrial septal defect; HFH = heart failure re-hospitalization; LAVI = left atrial volume endex; LVEDD = left ventricular end-diastolic diameter; LVEDV = left ventricular end diastolic volume; LVEF = left ventricular ejection fraction; M-TEER = mitral transcatheter edge-to-edge repair; MR = mitral regurgitation; MV = mitral valve; NYHA = New York Heart Association; SLDA = single leaflet device attachment; SPAP = systolic pulmonary artery pressure; SMR = secondary mitral regurgitation; TAPSE = tricuspid annular plane systolic excursion; TIA = transient ischemic attack: TMVI = transcatheter mitral valve implantation; TR = tricuspid regurgitation

**Supplementary Table 4**: Baseline characteristics and technical success according to availability of 1-year follow-up data

| Deceased patients excluded | **Patients with 1-year data (alive)**  N=152 | **Patients without 1-year data (lost to follow-up)**  N=128 | **P-value** |
| --- | --- | --- | --- |
| Age at M-TEER (years) | 81.9 ± 5.7 | 82.4 ± 7.0 | 0.492 |
| Female (%) | 74 (44.3) | 74 (48.6) | 0.441 |
| EuroSCORE II, % | 4.5 ± 4.1 | 5.6 ± 4.7 | 0.088 |
| STS Score for MV replacement, % | 3.9 ± 3.7 | 4.8 ± 3.9 | 0.087 |
| BMI (kg/m2) | 24.4 ± 4.2 | 24.3 ± 5.2 | 0.898 |
| Obesity (BMI ≥ 30 kg/m2) | 15 (9.0) | 20 (13.5) | 0.202 |
| Arterial hypertension | 117 (70.1) | 109 (74.1) | 0.421 |
| Severe renal failure (eGFR < 30 ml/min/1.73 m2) | 21 (12.6) | 31 (20.9) | 0.065 |
| Diabetes | 15 (9.0) | 21 (14.2) | 0.159 |
| Dyslipidemia | 61 (36.5) | 56 (37.8) | 0.810 |
| History of malignancy | 24 (22.0) | 32 (23.9) | 0.732 |
| Atrial fibrillation | 89 (53.3) | 74 (50.0) | 0.559 |
| Chronic obstructive pulmonary disease | 14 (8.4) | 17 (11.5) | 0.449 |
| Coronary artery disease | 59 (35.3) | 57 (38.5) | 0.559 |
| Prior myocardial infarction | 14 (8.4) | 15 (10.1) | 0.591 |
| Prior percutaneous coronary intervervention | 41 (24.6) | 42 (28.4) | 0.442 |
| History of Stroke | 14 (8.4) | 17 (11.7) | 0.356 |
| *Pulmonary Hypertension (mPAP ≥ 20 mmHg)  (N=106, 88 with follow-up and 45 without) | 69 (78.4) | 37 (82.2) | 0.605 |
| Anemia (Female: Hb < 120 g/L; Male: Hb < 130 g/L) | 76 (45.5) | 70 (47.3) | 0.751 |
| Previous TAVI | 5 (3.6) | 3 (3.8) | 0.942 |
| Previous SAVR | 7 (5.1) | 4 (5.1) | 0.995 |
| Previous surgical MV repair | 3 (1.8) | 4 (2.7) | 0.710 |
| Previous CABG | 12 (7.2) | 13 (8.8) | 0.678 |
| Heart failure hospitalization in the last 12 months prior to M-TEER  (N=143, 79 with follow-up and 64 without) | 20 (25.3) | 21 (32.8) | 0.324 |
| Creatinine (µmol/L) | 96 [78-176] | 98 [82-160] | 0.121 |
| NT-proBNP (pg/ml) | 2125 [962-3508] | 3735 [1663-7473] | 0.054 |
| Renin-angiotensin system inhibitor  ACE  ARB  Sacubitril-Valsartan | 53 (32.3)  57 (34.8)  4 (2.4) | 56 (37.8)  41 (27.7)  1 (0.7) | 0.298 |
| MRA | 14 (8.5) | 18 (12.5) | 0.268 |
| B-Blocker | 97 (59.1) | 88 (61.1) | 0.725 |
| Diuretics (excluding MRA) One agent Two agents | 132 (81.0)  3 (1.8) | 108 (76.1)  4 (2.8) | 0.557 |
| Loop diuretics dose  Furosemide equivalence dose (mg) | 40 [20-40] | 50 [20-80] | 0.324 |
|  |  |  |  |
| LVEF (%) | 58 ± 11 | 59 ± 10 | 0.214 |
| MR Severity  Moderate-to-severe (3+)  Severe (4+) | 26 (15.6)  141 (84.4) | 23 (15.5)  125 (84.5) | 0.994 |
| Lesion causing PMR  A2-P2 Prolapse/Flail | 67 (40.1) | 62 (41.9) | 0.819 |
| Complex MV anatomy | 52 (34.0) | 60 (41.4) | 0.191 |
| Technical success (MVARC definition) | 128 (93.4%) | 72 (92.3%) | 0.785 |

Results are expressed as absolute number (percentage) for categorical variables and mean (±SD) or median [interquartile range] for continuous variables.

* mPAP measured invasively

ACE = angiotensin-converting enzyme; ARB = angiotensin receptor blocker; BMI = body mass index; CABG = coronary artery bypass graft; eGFR = estimated glomerular filtration rate; Hb = haemoglobin; LVEF = left ventricular ejection fraction; MVARC = Mitral Valve Academic Research Consortium; mPAP = mean pulmonary artery pressure; MR = mitral regurgitation; MRA = mineralocorticoid receptor antagonist; M-TEER = mitral transcatheter edge-to-edge repair; MV = mitral valve; NYHA = New York Heart Association; PMR = primary mitral regurgitation; TAVI = transcatheter aortic valve implantation; SAVR = surgical aortic valve replacement; STS = Society of Thoracic Surgeons

**Supplementary Table 5**: Baseline characteristics in the subgroup of patients (N=143) whose anatomical complexity¶ could be analysed.

|  | **Non-Complex**  N=75 | **Complex**  N=68 | **P-value** |
| --- | --- | --- | --- |
| Age at M-TEER (years) | 82.7 ± 5.9 | 79.4 ± 7.0 | **0.003** |
| Female (%) | 37 (49.3) | 33 (48.5) | 0.923 |
| EuroSCORE II, % | 5.2 ± 4.1 | 7.3 ± 5.8 | 0.411 |
| STS Score for MV replacement, % | 5.1 ± 3.4 | 3.8 ± 3.0 | 0.580 |
| BMI (kg/m2) | 24.3 ± 4.5 | 24.8 ± 5.7 | 0.554 |
| Obesity (BMI ≥ 30 kg/m2) | 9 (12.0) | 9 (13.2) | 0.824 |
| Arterial hypertension | 54 (72.0) | 56 (82.3) | 0.099 |
| Severe renal failure (eGFR < 30 ml/min/1.73 m2) | 16 (21.3) | 11 (16.2) | 0.431 |
| Diabetes | 13 (17.3) | 4 (5.9) | **0.035** |
| Dyslipidemia | 34 (45.3) | 37 (54.4) | 0.287 |
| History of malignancy | 25 (33.3) | 13 (19.1) | 0.055 |
| Atrial fibrillation | 48 (64.0) | 42 (61.8) | 0.782 |
| Chronic obstructive pulmonary disease | 8 (10.7) | 8 (11.8) | 0.835 |
| Coronary artery disease | 18 (24.0) | 22 (32.4) | 0.266 |
| Prior myocardial infarction | 2 (2.7) | 8 (11.8) | **0.033** |
| Prior percutaneous coronary intervention | 11 (14.7) | 16 (23.5) | 0.176 |
| History of Stroke | 7 (9.3) | 8 (11.8) | 0.636 |
| *Pulmonary Hypertension (mPAP ≥ 20 mmHg)  (N=80, 49 in Non-Complex and 31 in Complex) | 45 (91.8) | 25 (80.6) | 0.140 |
| Anemia (Female: Hb < 120 g/L; Male: Hb < 130 g/L) | 44 (58.7) | 28 (41.2) | **0.037** |
| Previous TAVI | 1 (1.3) | 4 (5.9) | 0.139 |
| Previous SAVR | 3 (4.0) | 4 (5.9) | 0.602 |
| Previous surgical MV repair | 0 | 0 | NA |
| Previous CABG | 9 (12.0) | 4 (5.9) | 0.204 |
| Heart failure hospitalization in the last 12 months prior to M-TEER | 19 (25.3) | 22 (32.4) | 0.354 |
| NYHA functional class  I  II  III  IV | 9 (12.0)  21 (28.0) 41 (54.7) 4 (5.3) | 7 (10.3) 19 (27.9)  36 (52.9) 6 (8.8) | 0.865 |
| eGFR (ml/min) | 42 [32-57] | 51 [35-73] | **0.032** |
| Creatinine (µmol/L) | 100 [79-133] | 91 [76-110] | 0.067 |
| NT-proBNP (pg/ml) | 2590 [970 - 5439] | 1715 [591-4216] | 0.136 |
| Renin-angiotensin system inhibitor  ACE  ARB  Sacubitril-Valsartan | 26 (34.7)  25 (33.3)  2 (2.7) | 30 (44.1)  26 (38.2)  0 | 0.178 |
| MRA | 9 (12.0) | 11 (16.2) | 0.472 |
| B-Blocker | 46 (61.3) | 45 (66.2) | 0.548 |
| Diuretics (excluding MRA)  (N=140, 73 in Non-Complex and 67 in Complex) One agent Two agents | 56 (76.7)  6 (8.2) | 52 (77.6)  0 | **0.038** |
| Loop diuretics dose  Furosemide equivalence dose (mg) | 40 [20-80] | 40 [20-80] | 0.603 |

Results are expressed as absolute number (percentage) for categorical variables and mean (±SD) or median [interquartile range] for continuous variables.

¶Anatomical complexity defined as presence of ≥1 of the following criteria: ≥moderate calcifications, Barlow disease, multiple prolapses or commissural prolapse

* mPAP measured invasively

ACE = angiotensin-converting enzyme; ARB = angiotensin receptor blocker; BMI = body mass index; CABG = coronary artery bypass graft; eGFR = estimated glomerular filtration rate; Hb = haemoglobin; mPAP = mean pulmonary artery pressure; MR = mitral regurgitation; MRA = mineralocorticoid receptor antagonist; M-TEER = mitral transcatheter edge-to-edge repair; MV = mitral valve; NYHA = New York Heart Association; PMR = primary mitral regurgitation; TAVI = transcatheter aortic valve implantation; SAVR = surgical aortic valve replacement; STS = Society of Thoracic Surgeons

**Supplementary Table 6**: Last follow-up (median of 22 months [9-36], minimum 0 and maximum 72) outcomes after M-TEER in the subgroup of patients (N=143) whose anatomical complexity¶ could be analysed.

|  | **Non-Complex**  N=75 | **Complex**  N=68 | **P-value** |
| --- | --- | --- | --- |
| Residual MR severity (N=119, 64 in Non-Complex and 55 in Complex)  None/trace (0)  Mild (1+)  Moderate (2+)  Moderate-to-severe (3+)  Severe (4+) | 4 (6.3)  26 (40.6)  21 (32.8)  6 (9.4)  7 (10.9) | 3 (5.5)  16 (29.1)  21 (38.2)  5 (9.1)  10 (18.2) | 0.649 |
| Residual MR ≤ Mild | 39 (46.9) | 19 (34.5) | 0.173 |
| Mean transvalvular MV pressure gradient (mmHg) | 4 [3-4] | 4 [3-5] | 0.172 |
| LVEF (%) | 54 ± 10 | 51 ± 12 | 0.966 |
| LVEDV (ml) | 111 ± 38 | 122 ± 65 | 0.444 |
| LVEDD (mm) | 50 ± 9 | 50 ± 9 | 0.980 |
| LAVI biplane (ml/m2) | 82 ± 29 | 76 ± 45 | 0.564 |
| TAPSE (mm) | 20 ± 5 | 17 ± 6 | 0.078 |
| Estimated SPAP (mmHg) | 44 [35-53] | 43 [31-49] | 0.182 |
| TR severity (N=111, 58 in Non-Complex and 53 in Complex)  None/trace  Mild  Moderate  Severe  Massive  Torrential | 3 (5.2)  29 (50.0)  14 (24.1)  12 (20.7)  0  0 | 2 (3.8)  25 (47.2)  20 (37.7)  6 (11.3)  0  0 | 0.343 |
| NYHA functional class  (N=100, 55 in Non-Complex and 45 in Complex)  I  II  III  IV | 10 (18.2)  33 (60.0)  9 (16.4)  3 (5.5) | 5 (11.1)  24 (53.3)  14 (31.1)  2 (4.4) | 0.333 |
| Stroke (N=143, 75 in Non-Complex and 68 in Complex) | 4 (5.3) | 3 (4.4) | 0.671 |
| TIA (N=143, 75 in Non-Complex and 68 in Complex) | 1 (1.3) | 1 (1.5) | 0.677 |
| Myocardial infarction  (N=143, 75 in Non-Complex and 68 in Complex) | 1 (1.3) | 0 | 0.339 |
| Bleeding complications  (N=143, 75 in Non-Complex and 68 in Complex)  None  Minor  Major  Life-threatening  Fatal | 1 (1.3)  74 (98.7)  0  1 (1.3)  0  0 | 2 (2.9)  66 (97.1)  2 (2.9)  0  0  0 | 0.210 |
| Heart failure re-hospitalization  (N=143, 75 in Non-Complex and 68 in Complex) | 6 (8.0) | 3 (4.4) | 0.378 |
| SLDA  (N=119, 64 in Non-Complex and 55 in Complex) | 3 (4.7) | 2 (3.6) | 0.776 |
| MV re-intervention  (N=143, 75 in Non-Complex and 68 in Complex)  Type of MV re-intervention  Elasta-Clip followed by transapical TMVI | 3 (4.0)  3 (4.0) | 0  0 | 0.096 |
| All-cause mortality  (N=143, 75 in Non-Complex and 68 in Complex) | 26 (34.7) | 35 (51.5) | **0.042** |

Results are expressed as absolute number (percentage) for categorical variables and mean (±SD) or median [interquartile range] for continuous variables.

¶Anatomical complexity defined as presence of ≥1 of the following criteria: ≥moderate calcifications, Barlow disease, multiple prolapses or commissural prolapse

LAVI = left atrial volume endex; LVEDD = left ventricular end-diastolic diameter; LVEDV = left ventricular end diastolic volume; LVEF = left ventricular ejection fraction; M-TEER = mitral transcatheter edge-to-edge repair; MR = mitral regurgitation; MV = mitral valve; SLDA = single leaflet device attachment; SPAP = systolic pulmonary artery pressure; SMR = secondary mitral regurgitation; TAPSE = tricuspid annular plane systolic excursion; TIA = transient ischemic attack: TMVI = transcatheter mitral valve implantation; TR = tricuspid regurgitation
